# *Hanseniaspora smithiae* sp. nov., a Novel Apiculate Yeast Species From Patagonian Forests That Lacks the Typical Genomic Domestication Signatures for Fermentative Environments

**DOI:** 10.3389/fmicb.2021.679894

**Published:** 2021-07-21

**Authors:** Neža Čadež, Nicolas Bellora, Ricardo Ulloa, Miha Tome, Hrvoje Petković, Marizeth Groenewald, Chris Todd Hittinger, Diego Libkind

**Affiliations:** ^1^Food Science and Technology Department, Biotechnical Faculty, University of Ljubljana, Ljubljana, Slovenia; ^2^Centro de Referencia en Levaduras y Tecnología Cervecera (CRELTEC), Instituto Andino Patagónico de Tecnologías Biológicas y Geoambientales (IPATEC), Consejo Nacional de Investigaciones, Científicas y Técnicas (CONICET), Universidad Nacional del Comahue, Bariloche, Argentina; ^3^Laboratorio de Bioprocesos, Instituto de Investigación y Desarrollo en Ingeniería de Procesos, Biotecnología y Energías Alternativas, Consejo Nacional de Investigaciones, Científicas y Técnicas (CONICET), Universidad Nacional del Comahue, Neuquén, Argentina; ^4^Westerdijk Fungal Biodiversity Institute, Utrecht, Netherlands; ^5^Laboratory of Genetics, Center for Genomic Science Innovation, DOE Great Lakes Bioenergy Research Center, Wisconsin Energy Institute, J. F. Crow Institute for the Study of Evolution, University of Wisconsin-Madison, Madison, WI, United States

**Keywords:** apiculate yeast, new species, phylogenomics, OGRI, gene loss, speciation, domestication

## Abstract

During a survey of *Nothofagus* trees and their parasitic fungi in Andean Patagonia (Argentina), genetically distinct strains of *Hanseniaspora* were obtained from the sugar-containing stromata of parasitic *Cyttaria* spp. Phylogenetic analyses based on the single-gene sequences (encoding rRNA and actin) or on conserved, single-copy, orthologous genes from genome sequence assemblies revealed that these strains represent a new species closely related to *Hanseniaspora valbyensis.* Additionally, delimitation of this novel species was supported by genetic distance calculations using overall genome relatedness indices (OGRI) between the novel taxon and its closest relatives. To better understand the mode of speciation in *Hanseniaspora*, we examined genes that were retained or lost in the novel species in comparison to its closest relatives. These analyses show that, during diversification, this novel species and its closest relatives, *H. valbyensis* and *Hanseniaspora jakobsenii*, lost mitochondrial and other genes involved in the generation of precursor metabolites and energy, which could explain their slower growth and higher ethanol yields under aerobic conditions. Similarly, *Hanseniaspora mollemarum* lost the ability to sporulate, along with genes that are involved in meiosis and mating. Based on these findings, a formal description of the novel yeast species *Hanseniaspora smithiae* sp. nov. is proposed, with CRUB 1602^*H*^ as the holotype.

## Introduction

The yeasts of the genus *Hanseniaspora* (Saccharomycodaceae, Ascomycota) are characterized by their apiculate form, bipolar budding, and basal phylogenetic placement relative to the family Saccharomycetaceae. Currently there are 20 recognized taxa of *Hanseniaspora*, including three recently assigned anamorphic species formerly known as *Kloeckera* spp. (Čadež et al., 2019). The latest incorporations to the genus were *Hanseniaspora mollemarum* from The Netherlands (Groenewald et al., 2018), *Hanseniaspora gamundiae* from Argentina (Čadež et al., 2019), and *Hanseniaspora terricola* from Tibet (Liu et al., 2021). Species of *Hanseniaspora* are widespread and particularly abundant on various fruits, as well as on flowers and bark, which are their primary habitats (Saubin et al., 2020). The vectors for their dispersal are mostly insects, such as *Drosophila* spp., which are attracted by the aromatic volatile compounds produced by these yeasts (Hamby et al., 2012; Becher et al., 2018). Due to their ability to ferment simple sugars, they contribute to the beginning phases of various spontaneous food fermentation processes, and thus, many *Hanseniaspora* spp. have been intensively studied to determine their potential to improve sensorial complexity of fermented products (Steensels and Verstrepen, 2014), primarily for wine (Martin et al., 2018), but more recently for beer production (Burini et al., 2021). Strains of the species *Hanseniaspora uvarum, Hanseniaspora guilliermondii, Hanseniaspora viniae*, and *Hanseniaspora valbyensis* are probably the most relevant for bioflavoring in the beverage industry.

Recently, it was shown that species of the genus *Hanseniaspora* have evolved unusually rapidly, likely due to the loss of many genes associated with DNA repair and maintenance (Steenwyk et al., 2019). Increased mutation rates can accelerate adaptation to fluctuating and stressful conditions (Giraud et al., 2001; Healey et al., 2016) and represent one of the drivers of intrinsic reproductive isolation among diverging populations (Seehausen et al., 2014). Furthermore, the observed pervasive gene loss in *Hanseniaspora* was shown to have a biased pattern in the functional categories of metabolism and the cell cycle. This bias might be associated with the lifestyle of these yeasts, as they are dominant on ripe fruit where simple sugars are available as a food source for only one period each year. *Hanseniaspora* species may have adapted to novel environments of flowering plants that produce sugar-based fruit once a year by the conditional dispensability of genes that are not required under specific conditions. At the same time, the loss of cell-cycle checkpoint genes may have led to rapid growth as an advantage over competitors.

To gain insight into the ecological causes of gene loss during adaptation of *Hanseniaspora* species to novel ecological niches, we continue our exploration of the yeast communities associated with *Nothofagus* trees and its parasitic fungi in Andean Patagonia. We discovered a group of apiculate yeast strains that are genetically distinct from their sister species *H. valbyensis* based on rRNA gene sequence and genomic data. In the present paper, we formally describe a novel species of apiculate yeast where their delineation is additionally based on data of complete genomic sequences of 20 type strains of *Hanseniaspora* species, two of which were sequenced during the present study. The genomic contents of the new species and its closest relatives, *H. valbyensis, H. mollemarum*, *Hanseniaspora lindneri, Hanseniaspora singularis*, and *Hanseniaspora jakobsenii*, correlate with their physiological and ecological backgrounds.

## Materials and Methods

### Yeast Isolation and Phenotypic Characterization

The strains examined in this study are listed in Supplementary Table 1. These included five isolates from the stromata of *Cyttaria* species and the bark of *Nothofagus betuloides* and *Nothofagus antarctica*, which were collected from sites very distant from each other (ca. 2,000 km) in the Andean forests in Patagonia, Argentina. Isolations were obtained according to the enrichment protocol described by Sampaio and Gonçalves (2008) and Libkind et al. (2011a). The Argentinean National Park Administration issued permission to Diego Libkind for this sampling. The strains were phenotypically characterized by methods described by Kurtzman et al. (2011). Assimilation tests were performed in liquid media following standard procedures. Sporulation was investigated on 2% malt extract (BD Difco) and 5% malt extract (Sigma) agars at 25°C, and cells were examined weekly for up to 4 weeks.

### DNA Extraction, Amplification, and Sequencing

MasterPure Yeast DNA Purification kits (Lucigen) were used to extract DNA from cultures grown on yeast extract–peptone–glucose (YPD, Conda) agar plates for 2 days. Amplification of DNA sequences that encode the internal transcribed spacer (ITS; 668 bp), the large subunit rRNA (LSU) D1/D2 domain (573 bp), and actin (encoded by *ACT1*; 949 bp) was performed as described by Cadez et al. (2006). These DNA sequences were determined using a commercial sequencing facility (Macrogen Inc., Netherlands). The sequences were assembled in BioNumerics 7.6 and aligned using the MEGA X software (Kumar et al., 2018) with the built-in MUSCLE algorithm (Edgar, 2004).

### Phylogenetic Analyses

Phylogenetic relationships among the five investigated yeast strains and their closest relatives were inferred using the maximum likelihood (ML) method with the substitution model of the Kimura two-parameter model calculated for actin, ITS, and the LSU D1/D2 concatenated datasets. The basic maximum parsimony network (Huson and Bryant, 2006), which finds the most parsimonious network using simulated annealing, was chosen using BioNumerics 7.6. Bootstrap support was determined from 1,000 pseudoreplicates (Felsenstein, 1985). For formal delineation of the phylogenetic species, a parsimony network using aligned ITS-D1/D2 sequences, excluding gaps at a 95% connection limit, was constructed using the TCS 1.21 program (Clement et al., 2000).

### Genome Sequencing, Assembly, and Annotation

The genomes of almost all type strains of *Hanseniaspora* species are publicly available, including the type strain of *Hanseniaspora smithiae* (Shen et al., 2018; Steenwyk et al., 2019). In the course of the present study, the genomes of *H. mollemarum* CBS 15034^T^ and *H. lindneri* CBS 285^T^ were sequenced. The genomic DNA was isolated according to a protocol published by Schwartz and Sherlock (2016) that is based on protein removal using phenol:chloroform. A sequencing library was constructed using TruSeq DNA PCR Free (350) kits (Illumina), and run on an Illumina NovaSeq instrument at the Macrogen Inc. sequencing facility.

To generate the whole-genome assembly, the Illumina reads were used as input to the meta-assembler pipeline of iWGS v1.1 (Zhou et al., 2016). The pipeline first trimmed the adapters and low-quality bases using Trimmomatic v.038 (Bolger et al., 2014), and calculated the optimal *k-*mer length for a range of assemblers. The quality of each assembly was evaluated by N_50_ statistics and genome size, using Quast v5.0.2 (Gurevich et al., 2013). The assembly produced by SPAdes v.3.11.1 (Bankevich et al., 2012) gave the best results, and was used further. The completeness of the genome was evaluated using the BUSCO v3.0.2 software (Simão et al., 2015).

For annotation of the *H. mollemarum* genome, the MAKER genome annotation pipeline v3.01.02 was used (Holt and Yandell, 2011). For the homology evidence for genome annotation, the proteomes and transcripts of *Saccharomyces cerevisiae, H. valbyensis*, and *H. uvarum* were used. To soft-mask repeats, RepeatMasker was used with the fungal RepBase repeat library. Furthermore, three *ab initio* gene predictions were used with the MAKER pipeline: SNAP (Korf, 2004), GeneMark-ES v4.57 (Ter-Hovhannisyan et al., 2008) trained for the *H. mollemarum* or *H. lindneri* genomes, and AUGUSTUS v3.3.2 (Stanke et al., 2008) with “*Saccharomyces*” as an Augustus pre-existing species model. All of the resulting gene models, together with the homology evidence and masked repeats, were used to perform the final set of annotations of the genome.

### Phylogenomic Placement and Genomic Distance Calculations

For the phylogenomic reconstruction of the genus *Hanseniaspora*, protein sequences of conserved single-copy orthologs were identified by BUSCO v3.0.2 using the “Saccharomycetales” reference dataset. A total of 251 conserved genes present in all *Hanseniaspora* species were extracted, aligned and trimmed using MAFFT v7.310 (Katoh and Standley, 2013) and trimAl v1.4 (Capella-Gutiérrez et al., 2009), which are part of the BUSCO USECASE genomic utilities pipeline (Waterhouse et al., 2018). The alignments were concatenated into a single data matrix that was used as input for ML phylogenetic inference with RAxML v8.2.11 (Stamatakis, 2014). To determine the best-fit phylogenetic model, the ModelFinder option of IQ-TREE v. 1.6.1 was used (Kalyaanamoorthy et al., 2017). Branch support was evaluated with 100 rapid bootstrap inferences, while internode certainty (IC) was used to evaluate the degree of conflicting bipartitions among the 251 individual gene trees (Salichos and Rokas, 2013; Salichos et al., 2014); both were carried out in RAxML. For calculations, the ML gene-based trees were constructed in RAxML with the best-fit model of amino-acid substitutions that were separately estimated for each gene dataset in IQ-TREE. The gene-based partitions were used to calculate the IC values on the concatenated gene tree in RAxML (option –f i).

Similarities between the genomes of *Hanseniaspora* species were calculated using average nucleotide identity (ANI) metrics with the web-based calculator available at https://www.ezbiocloud.net/tools/ani (Yoon et al., 2017) and with command-line tool FastANI (Jain et al., 2018). To calculate distances between genomes, digital DNA-DNA homology (dDDH) values were calculated using the Genome-to-Genome Distance Calculator 2.1, provided by the German Collection of Microorganisms and Cell Cultures website^1^. The dDDH values presented here were calculated using Formula 2, which estimates them based on the identities of high-scoring segment pairs (Meier-Kolthoff et al., 2013). Heatmap visualization and clustering based on dDDH and ANI values were created using Orange v3.23.1 (Demšar et al., 2013).

### Genome Searches for Genes Involved in Sugar Assimilation

To determine the presence or absence and the copy number of genes related to carbon source assimilation, their profiles in the genomes of *H. smithiae* and its closest relatives were defined using the National Center for Biotechnology Information BLAST + v2.8.1 BLASTP and TBLASTX tools with query protein sequences from *S. cerevisiae* S288C (all proteins except Lac4 and Lac12) and *Kluyveromyces marxianus* DMKU3-1042 (Lac4 and Lac12) against subject databases built from the *Hanseniaspora* proteomes and transcriptomes. The *E*-value threshold was set to 10^–10^ to assign copy numbers of each gene as described by Haase et al. (2017).

### Screening for Gained and Lost Genes in the Novel Species

Steenwyk et al. (2019) reported the presence and absence of *S. cerevisiae* homologs in *Hanseniaspora* genomes. The results are available from figshare: https://doi.org/10.6084/m9.figshare.7670756.v2. However, since the genomes of *H. mollemarum* and *H. smithiae* were sequenced later, we searched for the genes using a hidden Markov model, as described in detail by Steenwyk et al. (2019).

The matrix of present/absent orthologs of *H. smithiae* and five of its closest relatives was imported into Orange v3.23.1 (Demšar et al., 2013) for data mining, in which a Venn diagram was used to select the unique genes present in *H. smithiae* and its closest relatives. To determine in which functional categories these genes clustered, we conducted a functional grouping of genes based on gene ontology (GO) annotations (Ashburner et al., 2000; The Gene Ontology Consortium, 2019), using the “Yeast” subset on the *Saccharomyces* Genome Database (Cherry et al., 2012). To further visualize the species-specific GO annotations, we built a GO network using REViGO (Supek et al., 2011) with the following settings: medium list size (0.7) with SimRel semantic similarity measures to calculate the *p*-values.

### Growth Kinetics and Ethanol Production Under Aerobic Conditions

Growth was followed manually under semi-aerobic conditions in shake flasks on a rotary shaker (Multitron, Infors) in triplicates. First, overnight cultures suspended in saline solution were inoculated to OD 0.1 in 50 ml YPD (Conda) in 250 ml shake flasks and cultivated on an orbital shaker at high intensity (220 rpm) at 28°C for 24 h. During the exponential growth phase, optical density measurements were taken manually at time intervals of 1–2 h at 600 nm on the Spark microplate reader (Tecan).

At the end of the exponential phase, 1 ml cell suspension was filtered through 0.2 μm filters for HPLC analysis to determine ethanol production and sugar consumption. Filtered samples were analyzed using an UltiMate 3000 HPLC system (Thermo Fisher) with 5 mM H_2_SO_4_ in water as the mobile phase at a flow rate of 0.6 ml/min. Relevant standards for quantification were prepared with ethanol (Merck, for analysis, ACS, ISO, Reag. Ph Eur, 100%) and glucose (anhydrous for biochemistry Reag. Ph Eur).

## Results and Discussion

### Species Boundaries and Phylogenetic Placement

**FIGURE 1:**
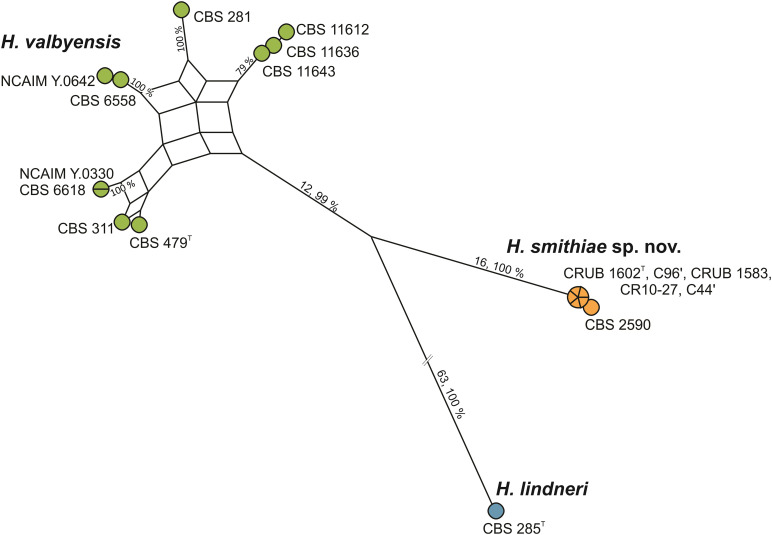
Maximum parsimony network for *H. smithiae* (6 strains), *H. valbyensis* (9 strains), and *H. lindneri* (one strain) based on the concatenated sequences of the actin gene and of the ITS and D1/D2 regions of rRNA (2,190 bp). Boxes (nodes subtended by multiple edges) indicate incongruence in the data. The numbers on the branches show the number of nucleotide substitutions and the bootstrap values (%).

During an exploration of fermentative yeast diversity in the natural Andean forests in Patagonia, Argentina, from 2006 to 2014, we collected ca. 800 samples of bark, soil, and stromata of the parasitic *Cyttaria* species of fungi that are associated with *Nothofagus* species of trees (Libkind et al., 2007, 2011a,b; Almeida et al., 2014; Eizaguirre et al., 2018). Although *Saccharomyces eubayanus* and *Saccharomyces uvarum* prevailed among the fermentative yeasts, non-*Saccharomyces* species of *Lachancea, Kregervanrija, Torulaspora, Zygosaccharomyces, Phaffia*, and *Hanseniaspora* were occasionally isolated as well. For *Hanseniaspora*, two strains that were collected from stromata of *Cyttaria* were recently formally described as *Hanseniaspora gamundiae* (Čadež et al., 2019). Furthermore, identification based on sequence comparisons of the LSU D1/D2 gene and the ITS sequences revealed that an additional five apiculate strains from *Cyttaria* stromata and *Nothofagus* tree bark were genetically divergent, as they differed by 5 (0.9% sequence divergence) and 11 (1.7% sequence divergence) nucleotide substitutions, respectively, from their closest relative of *H. valbyensis*. As the guidelines of Kurtzman and Robnett, 1998, which suggest that 1% substitutions in the LSU D1/D2 domain represent separate species, have been found to differ for closely related species of this rapidly evolving genus (Cadez et al., 2003, 2006), we searched for genetic discontinuities that would indicate interruption of gene flow between the species (Lachance et al., 2010). Operationally, based on the concatenated sequences of the actin gene (*ACT1)* and the ITS and LSU D1/D2 regions of rRNA, we constructed a maximum parsimony network (Figure 1) of five strains isolated from *Cyttaria*, the type strain of *H. lindneri*, and 10 strains from different collections (CBS, NCAIM) and from recent surveys in South Africa that were previously identified as *H. valbyensis*. The network analysis revealed reticulation that originated from incongruence between single gene trees (Supplementary Figure 1) between nine of the 10 *H. valbyensis* strains, which likely indicates gene flow within *H. valbyensis* (Lee et al., 2020). However, the five strains from *Cyttaria* and strain CBS 2590 (isolated from English draft beer and identified as *H. valbyensis* based on DNA-DNA reassociation measurements; Meyer et al., 1978) showed clear genetic discontinuities from their closest relatives. Based on these data, to accommodate these strains, we propose the novel species *Hanseniaspora smithiae* sp. nov.

**FIGURE 2:**
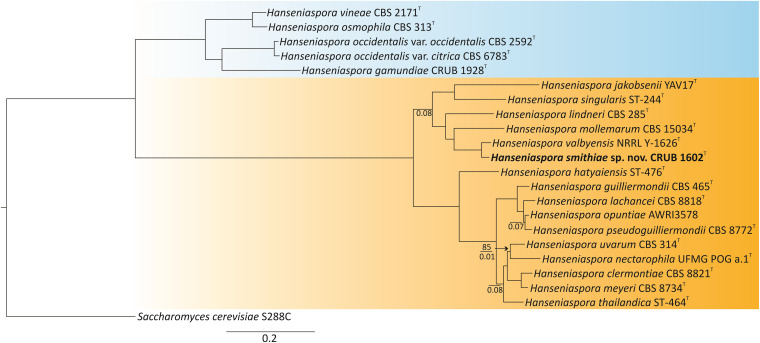
Phylogenomic tree showing the placement of the novel species *Hanseniaspora smithiae* sp. nov. CRUB 1602^T^. The ML tree was constructed based on a concatenated alignment of 251 orthologous, single-copy BUSCO proteins. Bootstrap values are shown only on branches where it is lower than 100% (above the line), while internode certainty (IC) values below 0.1 are shown below the line. Slower-evolving (SEL) and faster-evolving (FEL) lineages, according to Steenwyk et al. (2019), are colored in blue and orange, respectively. *Saccharomyces cerevisiae* was used as the outgroup.

To phylogenetically place *H. smithiae* within the genus, we selected the type or representative strains from each species of *Hanseniaspora* that had publicly available genome sequences (Riley et al., 2016; Steenwyk et al., 2019). The genomes of the type strains of the recently described species *H. mollemarum* (Groenewald et al., 2018) and of *H. lindneri* were sequenced in this study. Phylogenetic reconstruction was based on concatenated protein sequences of 251 conserved single-copy BUSCO orthologs, which yielded a tree fully consistent with the dichotomous tree of slower- and faster-evolving lineages (SEL and FEL, respectively) of Steenwyk et al. (2019) and placed *H. smithiae* within the faster-evolving lineage and sister to *H. valbyensis* (Figure 2).

Before genomic sequences of the type strains were available, the phylogeny of the closely related *Hanseniaspora* species of the FEL was poorly resolved, which was evident from the low bootstrap values (Čadež et al., 2019). However, because concatenation of a large number of genes nearly always produces a phylogeny with high bootstrap support, as shown in Figure 2, Salichos and Rokas (2013) suggested the use of internode certainty (IC) as an alternative measure to quantify congruence between the genes from which the phylogeny is reconstructed. All of the internodes had IC values > 0.1, which indicates that the majority of the individual gene tree topologies support the concatenated phylogeny. However, four out of 18 internodes had IC values < 0.1, which suggested considerable conflict among the gene trees. These conflicts were mostly concerned with the placement of *Hanseniaspora nectarophila* as sister to *H. uvarum* and within the species complex of *Hanseniaspora pseudoguilliermondii, Hanseniaspora opunitae*, and *Hanseniaspora lachancei.* The challenges in resolving this species complex may be because a lineage split into three descendant lineages at approximately the same time. Thus, the phylogenetic reconstruction of the species tree appears to be very difficult in this genus, which has highly variable rates of nucleotide substitution (Cadez et al., 2006; Steenwyk et al., 2019).

**FIGURE 3:**
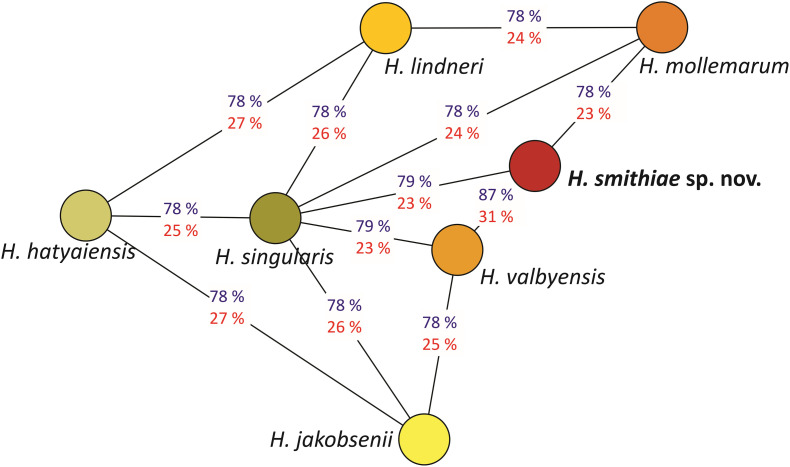
Genomic relationships between species closely related to *Hanseniaspora smithiae* sp. nov., based on the average nucleotide identity (ANI; blue) and the digital DNA-DNA homology values (red). The network was inferred using the minimum spanning tree algorithm on the ANI values.

The power of genomic data also lies in their use as tools that can calculate genome-wide genetic distances between a novel taxon and its closest relatives (Libkind et al., 2020b). For estimation of genetic distances between *H. smithiae* and its closest relatives (Figure 3), we used two tools that are available as web interfaces: the ANI calculator and the Genome-to-Genome Distance Calculator. The overall genome relatedness indices (OGRI) of the ANI and dDDH values of 87 and 31%, respectively, both confirmed the close relationship between *H. smithiae* and *H. valbyensis*, which is higher than between any of the other species of the *H. valbyensis* clade (ranges: 78–79% and 23–27%, respectively). To calibrate the utilities of the ANI and dDDH indices for delimiting species of a yeast genus for which an operational species concept was based on DNA reassociation values (Meyer et al., 1978), we arranged both types of values into distance matrices to reconstruct trees representing genomic relatedness within the genus (Supplementary Figures 2A,B). In contrast to dDDH, the ANI tree represents phylogenetic relationships between species very well, as also observed by Lachance et al. (2020) for the genus *Metschnikowia*. In general, we can divide the ANI -values into three categories: (1) between FEL and SEL (Figure 2), the values were below 77; (2) interspecific values within both lineages were between 77.3 and 81.1; and (3) between closely related species and varieties, values were between 83.2 and 91.8. In view of these results, we support the proposal of Libkind et al. (2020b) and Lachance et al. (2020) that OGRI values, preferably the ANI, should accompany any formal yeast species description.

### Gene Loss in *Hanseniaspora smithiae* and Its Relatives

**FIGURE 4:**
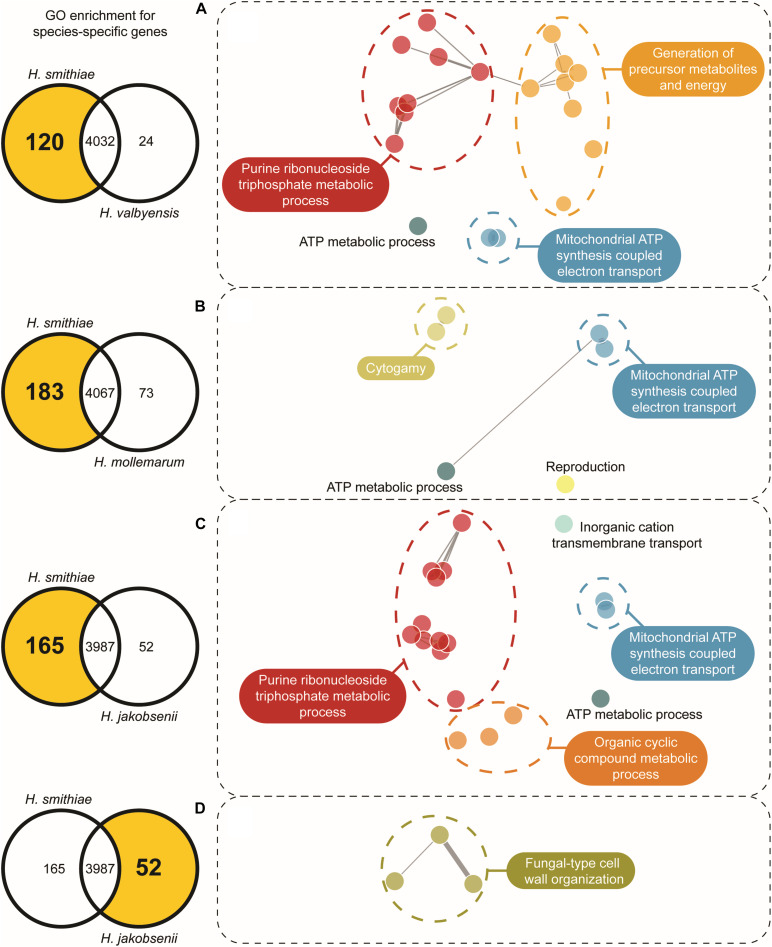
Gene ontology (GO) network based on enriched species-specific GO terms for biologic processes. The selected number of genes is indicated on the left side in the Venn diagram for each species comparison. For each highlighted zone of the Venn diagram in **(A–D)**, the GO networks were constructed using REViGO with clustering based on shared ontology groups, indicated by their unique color.

One of the main forces in the evolution of *Hanseniaspora* species has been their widespread loss of genes (Steenwyk et al., 2019). Therefore, we examined which genes have been retained or lost during speciation for *H. smithiae* in comparison to its closest relatives, *H. valbyensis*, *H. mollemarum*, *H. jakobsenii*, and *H. singularis*. From the list of genes present in the *H. smithiae*, *H. valbyensis*, *H. jakobsenii*, and *H. singularis* genomes obtained from Steenwyk et al. (2019) and the list of genes in the *H. mollemarum* genome prepared during this study, we performed comparative analyses of gene gains and losses using Venn diagrams following previously published procedures (Supplementary Figures 3A,B). In general, during speciation, *H. smithiae* retained more genes than its closest relatives. For example, *H. smithiae* lost 24 genes, but it retained 120 genes lost by its closest relative, *H. valbyensis*. To determine whether this phenomenon was biased toward specific groups of genes (Albalat and Cañestro, 2016), we performed gene ontology (GO) enrichment analysis (Figure 4 and Supplementary Table 2). The genes lost by *H. valbyensis* compared to *H. smithiae* were those involved in processes, such as the electron transport chain and ATP synthesis, as well as the generation of precursor metabolites and energy. It appears that, after branching apart from of *H. smithiae*, *H. valbyensis* lost mitochondrial and other genes involved in respiration, which could explain differences in growth rate and ethanol production during aerobic growth on fermentable sugar-rich media.

Therefore, we compared the specific growth rates, doubling times, and ethanol yields per cell of the two type strains of the species in exponential growth phase in rich medium (YPD) under semi-aerobic conditions (Table 1). Notably, *H. smithiae* grew significantly faster and produced less ethanol per cell than *H. valbyensis*, which is consistent with an impairment of respiratory metabolism by the latter. A similar observation was reported by Merico et al. (2007), who noted the absence of re-oxidation of NADH at the mitochondrial level in *H. valbyensis.* The present data suggest that *H. valbyensis* adapted to fermentative environments, resulting in the loss of many genes associated with respiration.

**TABLE 1:** Growth characteristics of *H. smithiae, H. valbyensis, H. jakobsenii*, and *S. cerevisiae*.

Species	Strain	μ_max_^a^ [h^–1^]	t_D_^b^ [h]	Υ _EtOH/glc_ per cell^c^ [fmol/cell]
*H. smithiae*	CRUB 1602^T^	0.45 ± 0.003	1.55 ± 0.01	14.06 ± 0.41
*H. valbyensis*	CBS 479^T^	0.39 ± 0.001	1.78 ± 0.01	34.98 ± 2.94
*H. jakobsenii*	ZIM 2603^T^	0.34 ± 0.009	2.05 ± 0.06	39.88 ± 3.68
*S. cerevisiae*	WLP001	0.31 ± 0.007	2.27 ± 0.06	85.70 ± 4.83

*^*a*^μ_*max*_ = maximal specific growth rate.*

*^*b*^t_*D*_ = doubling time.*

*^*c*^Υ_*E*__*tO*__*H/glc*_ = ethanol yield, the moles of ethanol produced from moles of glucose consumed.*

A similar but more profound loss was revealed by comparing the genes of *H. smithiae* and *H. jakobsenii*, a species isolated so far only from African palm wine in Burkina Faso (Ouoba et al., 2015). In comparison to *H. smithiae*, *H. jakobsenii* lost 95 genes associated with mitochondrial ATP synthesis and nuclear genes involved in the electron transport chain (Figure 4C). Similar to *H. valbyensis*, *H. jakobsenii* grew more slowly and produced higher amounts of ethanol when grown under aerobic conditions, suggesting that fermentative metabolism is a major source of energy for growth (Table 1). Furthermore, compared to *H. jakobsenii* (Figure 4D), the Patagonian species *H. smithiae* lost 10 genes involved in cell wall organization, most of which (7 of 10) belong to the *PAU* family, which are genes that are induced by anaerobiosis (Rachidi et al., 2000). These gene losses indicate a functional bias in the speciation of *H. smithiae*, which appears to be less adapted to man-made environments.

**FIGURE 5:**
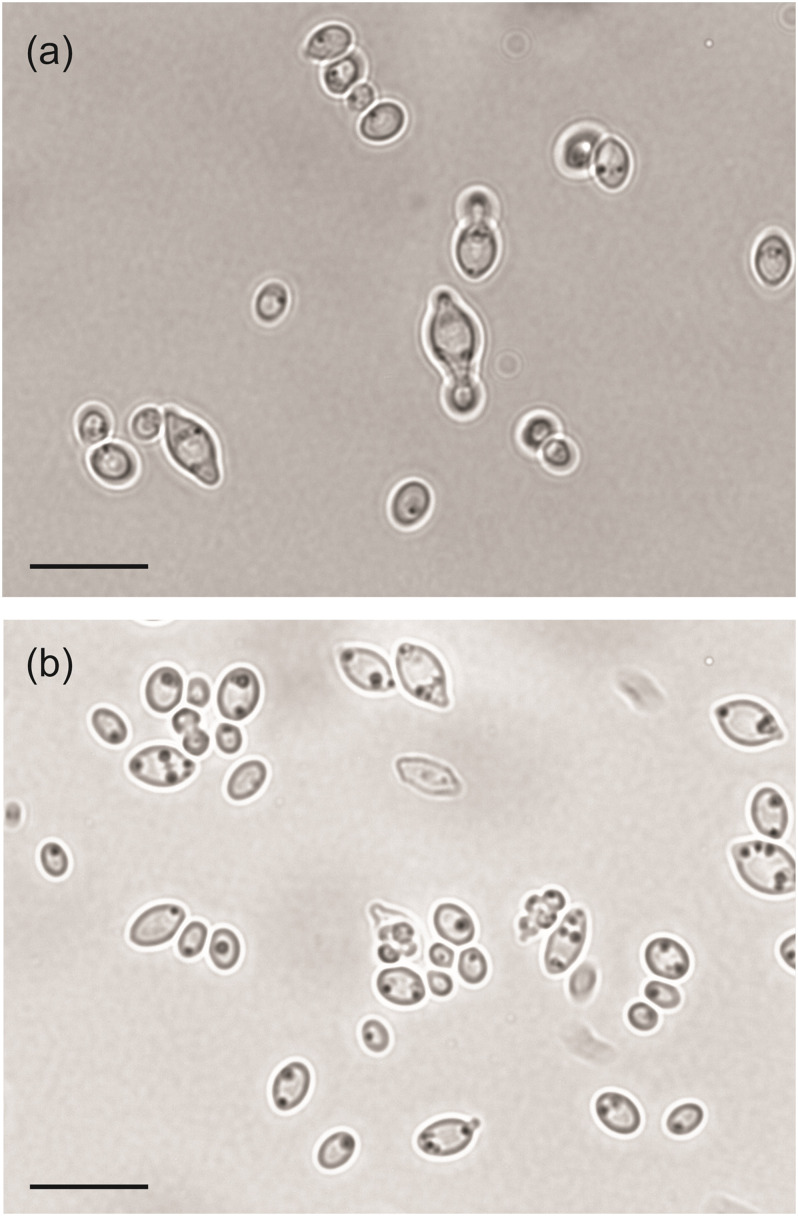
Microphotographs of *Hanseniapora smithiae* sp. nov. CRUB 1928^T^. **(a)** Budding cells; yeast–malt broth, 25°C, 220 rpm, 2 days. **(b)** Hat-to-helmet shaped ascospores released from the ascus; 5% malt extract agar, 14 days at 25°C. Scale bars, 10 μm.

We also examined the gene loss in *H. mollemarum* compared to *H. smithiae* (i.e., 85 lost genes). *H. mollemarum* is a recently described new species from Europe that was isolated from garden soil in The Netherlands, home-made elderberry syrup in Hungary, and home-made cider in England (Groenewald et al., 2018). In *H. mollemarum*, genes involved in the reproductive process are significantly overrepresented in the GO categories of lost genes (Figure 4B and Supplementary Table 2). Specifically, after mapping these to KEGG pathways, we found that many are involved in the MAP kinase intracellular signal transduction pathway, which is involved in responses to pheromones (e.g., via *FUS1*) and/or is associated in processes that coordinate cell cycle (e.g., via *BUB1, MAD3* and *MCD1*) and meiosis (e.g., via *SPO22* and *GIP1*). In addition, the mating type locus gene *MATα1* and a silenced mating cassette gene *HMLα1* were lost in *H. mollemarum*. The absence of these genes is most likely correlated with the inability of *H. mollemarum* to sporulate (Groenewald et al., 2018). Since *H. smithiae* is a homothallic species that producing two to four hat-shaped ascospores per ascus on malt extract agar (Figure 5), the loss of genes involved in meiosis could be a possible mechanism of genetic isolation of *H. mollemarum* from its ancestors.

Although it is particularly appealing to explain the process of speciation only by comparing the lists of genes that are present and absent between two species, the loss of genes is most likely not the only driver of their evolution. Nevertheless, most of the *Hanseniaspora* species are sexual diploids that can outcross, as shown recently by the discovery of interspecies hybrids isolated from various fruit in distinct regions (Saubin et al., 2020).

### Ecology and Correlation Between Phenotype and Genomic Content

Five of the six strains of *H. smithiae* were associated with parasitic fungi of *Cyttaria* on the *Nothofagus* beech tree, where they share their ecological niche with other fermentative yeasts, such as *S. uvarum*, *S. eubayanus* (Libkind et al., 2011a; Almeida et al., 2014; Eizaguirre et al., 2018), and *Phaffia rhodozyma* (Libkind et al., 2007, 2011b). Phenotypically, *H. smithiae* differs from its closest relative, *H. valbyensis*, in its ability to assimilate trehalose, albeit slowly and weakly. Trehalose is one of the sugars in the stromata of *Cyttaria* (Waksman, 1977), and this sugar is also assimilated by other sympatric species, such as *S. eubayanus*, although this ability varies among strains (Libkind et al., 2011a). Thus, the ability to assimilate trehalose might be one of the phenotypic advantages that allowed *H. smithiae* to thrive in stromata as its primary habitat.

**FIGURE 6:**
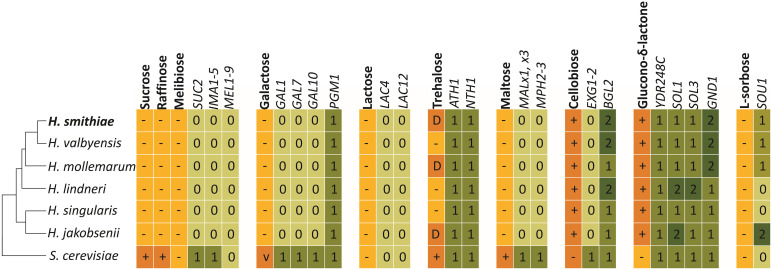
Metabolic traits and genes involved in the assimilation of carbon sources included in standard phenotypic characterization by *Hanseniaspora smithiae*, its closest relatives, and *Saccharomyce cerevisiae* (S288C). Numbered boxes indicate presence (1) or absence (0) of putative homologous genes, as determined using the BLASTP and TBLASTX tools.

To better understand the genetic causes of yeast metabolic diversity, we searched the *H. smithiae* genome for candidate genes that underlie the physiological traits used for traditional taxonomic identification. We analyzed the genome content of *H. smithiae* and its closest relatives for genes required for the use of disaccharides, as well as for the use of raffinose and glucono-δ-lactone (Figure 6). As noted previously, since *Hanseniaspora* species assimilate only a limited number of carbon sources, they also lack the genes required for the use of many carbon sources (Riley et al., 2016; Čadež et al., 2019; Steenwyk et al., 2019). Two notable exceptions are enzymes accurately predicting the utilization of cellobiose (*BGL2*) and glucono-δ-lactone. Another exception is for the genes predicted to encode acid trehalase (*ATH1*) and neutral trehalase (*NTH1*), two enzymes that are required for the utilization of extracellular trehalose (Jules et al., 2004). However, these genes are also present in *Hanseniaspora* species that cannot assimilate trehalose as their sole carbon source. This discordance is an additional example that illustrates that our knowledge of the genotype–phenotype relationships of non-conventional yeasts remains limited (Libkind et al., 2020a).

## Description of *Hanseniaspora Smithiae* Libkind, Čadež, Hittinger Sp. Nov

In recognition of Dr. Maudy Th. Smith (Netherlands), for her valuable contributions to *Hanseniaspora* taxonomy.

In yeast extract–malt extract liquid medium after 48 h at 25°C, the cells are apiculate, ovoid, or elongate (2.0–7.5) μm × (1.2–3.2) μm, and they occur singly or in pairs. Budding is bipolar. Sediment is present, and a very thin ring is formed after 1 month. On yeast extract–malt extract agar after 1 month at 25°C, the steak culture is cream-colored, butyrous, smooth, glossy and flat to slightly raised at the center, with an entire margin. Asci contain two to four hat-shaped to helmet-shaped ascospores (1.4–4.3 μm), which are usually released at maturity (Figure 5). Released ascospores usually aggregate. They were observed after 2 weeks or more on 5% malt extract agar (Difco) at 25°C. Glucose is fermented; D-galactose, maltose, lactose, and cellobiose are not fermented. The carbon compounds that are assimilated are glucose, α,α-trehalose, cellobiose, salicin, arbutin, glucono-δ-lactone, and D-gluconate; no growth occurs on galactose, L-sorbose, D-glucosamine, N-acetyl-D-glucosamine, D-ribose, D-xylose, L-arabinose, D-arabinose, L-rhamnose, sucrose, maltose, methyl α-D-glucoside, melibiose, lactose, raffinose, melezitose, inulin, starch, glycerol, erythritol, ribitol, xylitol, L-arabinitol, D-glucitol, D-mannitol, galactitol, *myo*-inositol, 2-keto-D-gluconate, D-glucuronate, D-galacturonate, DL-lactate, succinate, citrate, methanol, ethanol, propane-1,2-diol, butane-2,3-diol, and hexadecane. Assimilation of nitrogen compounds is positive for ethylamine hydrochloride, lysine, and cadaverine, but it is negative for potassium nitrate, sodium nitrite, creatine, creatinine, glucosamine, and imidazole. Growth in vitamin-free medium is absent. Growth occurs at 30°C, but it is absent at 35°C. Growth with 10% NaCl is positive, but it is absent with 16% NaCl, on 50% (w/w) glucose–yeast extract agar, with 1% acetic acid, and with 0.01% cycloheximide. The diazonium blue B reaction is negative.

The holotype (CRUB 1602^*H*^) is permanently maintained in a metabolically inactive state at the Centro Regional Universitario Bariloche Yeast Culture Collection, Argentina, and the type strain was deposited in the same collection (CRUB 1602^T^), in the ARS Culture Collection, National Center for Agricultural Utilization Research, IL, United States (NRRL Y-63759^T^), in the Portuguese Yeast Culture Collection, Caparica, Portugal (PYCC 7263^T^), and in the Collection of Industrial Microorganisms, Slovenia (ZIM 2538^T^). The strain CRUB 1602^T^ was isolated from *Cyttaria hariotii* stromata infecting *Nothofagus antarctica* in December 2006. The Mycobank number is MB839330. The BioProject number for raw genome sequencing reads is PRJNA529215 (BioSample SAMN11259523), and the GenBank accession number for the assembled genome is GCA_004919795.1 (Steenwyk et al., 2019).

## Data Availability Statement

The datasets presented in this study can be found in online repositories. The names of the repository/repositories and accession number(s) can be found below: https://www.mycobank.org/, MB839330. The GenBank accession numbers are listed in Supplementary Table 1.

## Author Contributions

NČ, MT, DL, and CH conceived the study and wrote the manuscript. NČ, MT, and NB performed the experiments and analyzed the data. RU and MG isolated the cultures. NČ and MT prepared the figures. All authors have reviewed and edited the manuscript.

## Conflict of Interest

The authors declare that the research was conducted in the absence of any commercial or financial relationships that could be construed as a potential conflict of interest.

## Footnotes

1 http://ggdc.dsmz.de/ggdc.php
