# Supplementary material for: Hanseniaspora smithiae sp. nov., a Novel Apiculate Yeast Species From Patagonian Forests That Lacks the Typical Genomic Domestication Signatures for Fermentative Environments
Source: Front Microbiol. 2021 Jul 21;12:679894. doi: 10.3389/fmicb.2021.679894 (PMC8334367; doi:10.3389/fmicb.2021.679894)
Supplement: Supplementary file 1 [file Data_Sheet_1.zip › Supplementary Material Figures.docx]

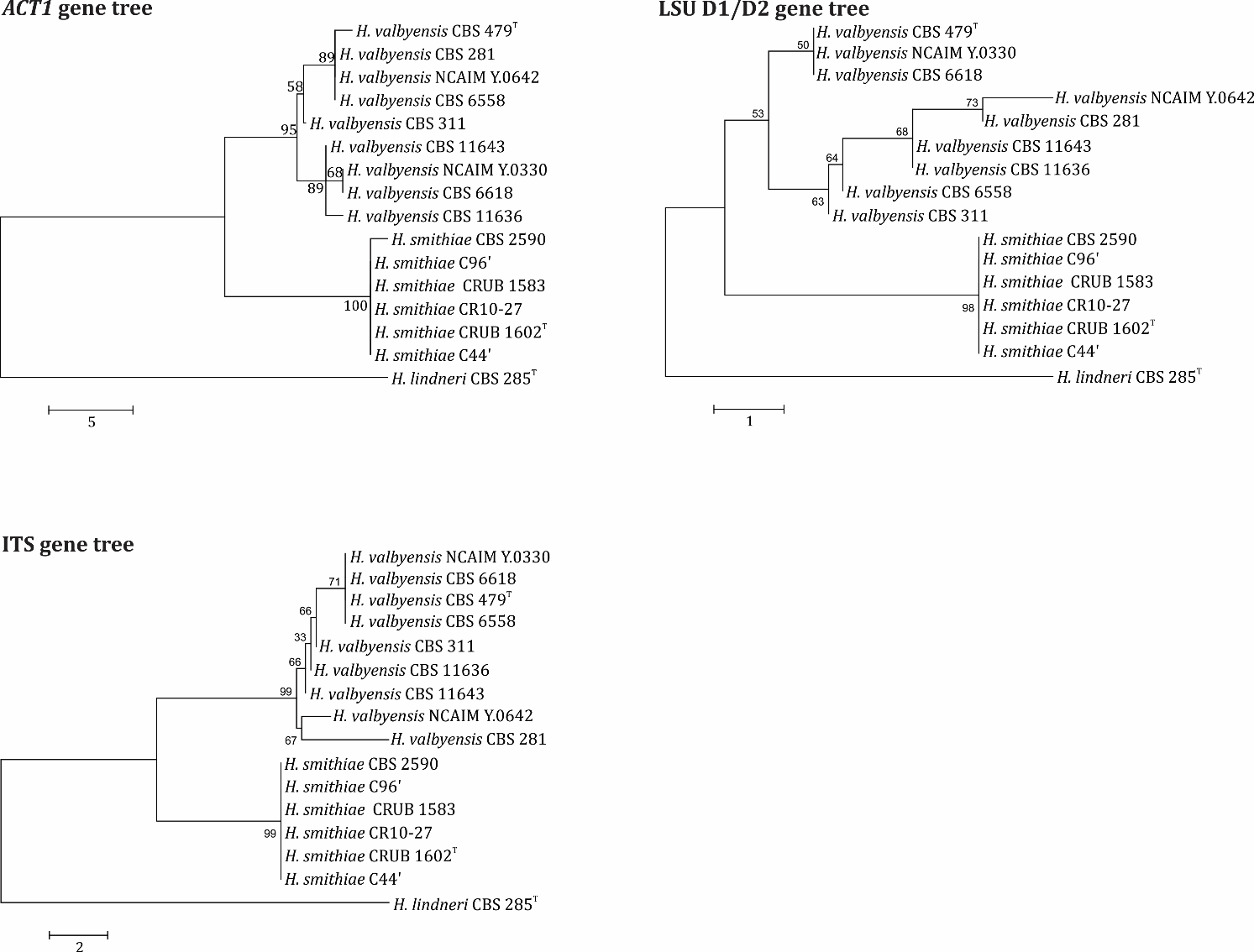


**Supplementary Figure S1|** Gene trees after analysis of the sequences of the actin gene, the LSU D1/D2 region, and the ITS region of *H. smithiae* and *H. valbyensis* strains using the neighbour-joining method and the maximum composite likelihood model of sequence evolution. Bootstrap percentages above 50% from 1,000 replicates are shown. The trees were rooted with *H. lindneri.* Branch lengths correspond to the number of base differences per sequence.


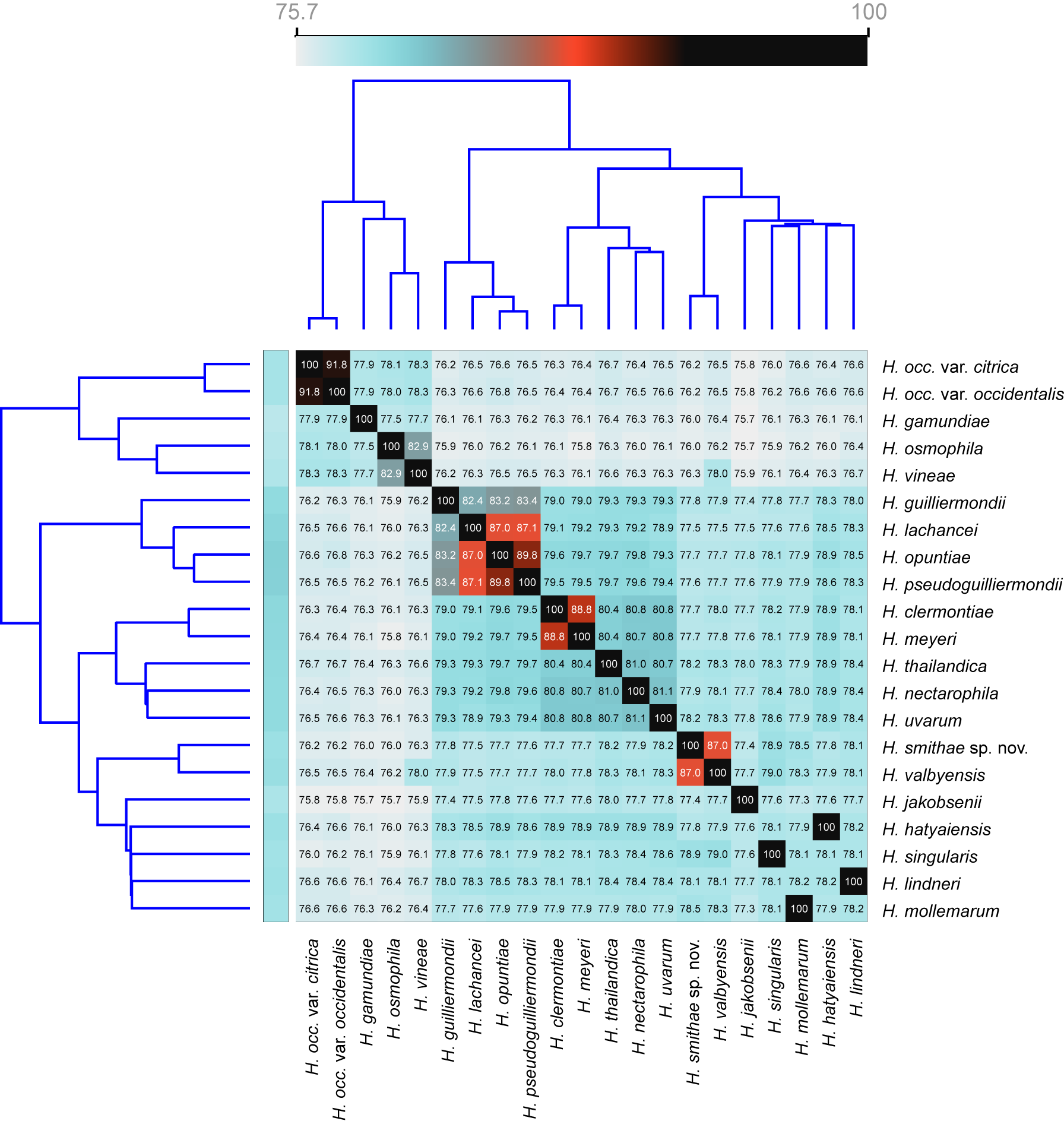


**Supplementary Figure 2A|** Cluster analysis and heatmap based on ANI values among *Hanseniaspora* species.

**
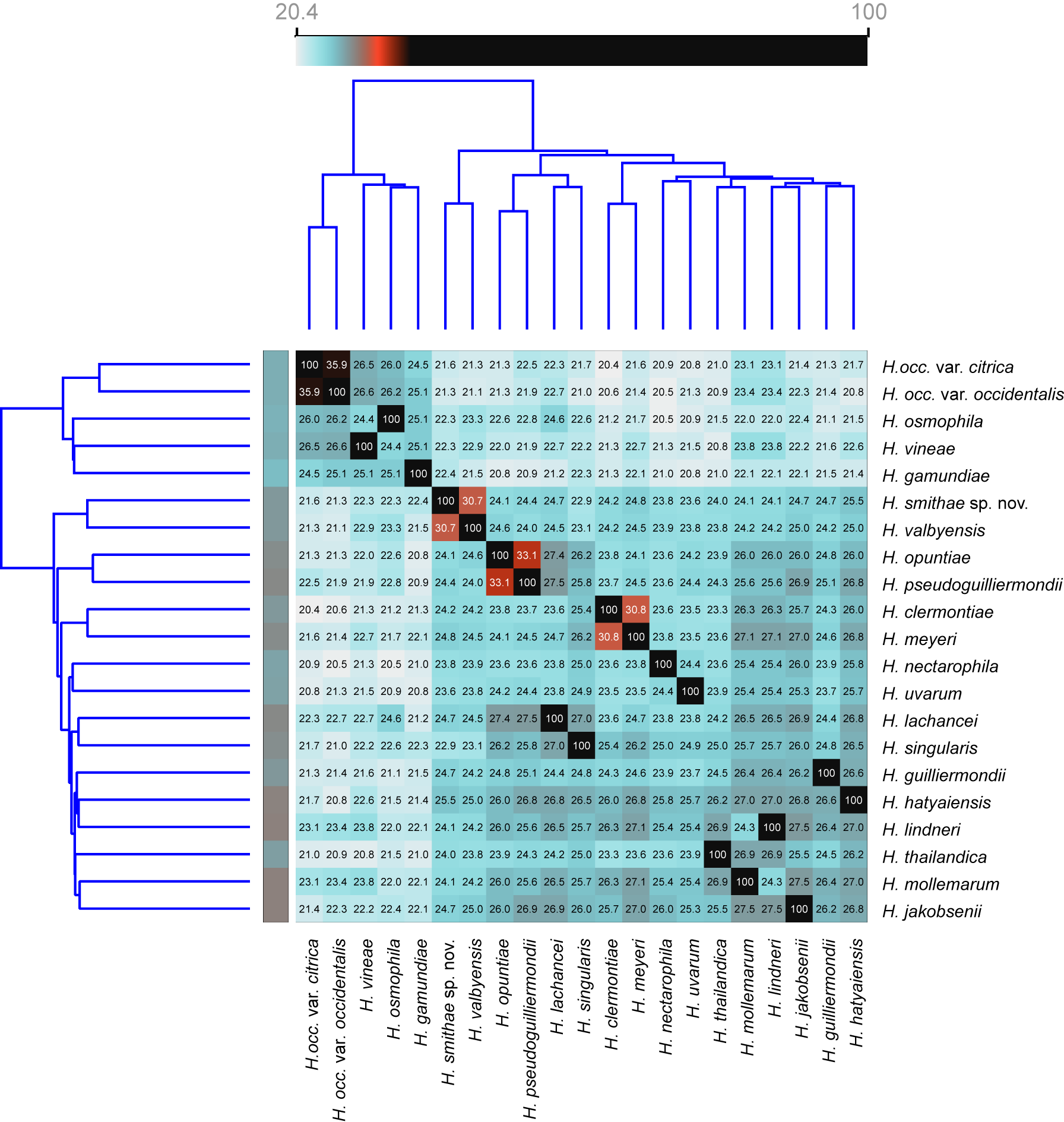
**

**Supplementary Figure 2B|** Cluster analysis and heatmap based on dDDH values among *Hanseniaspora* species.


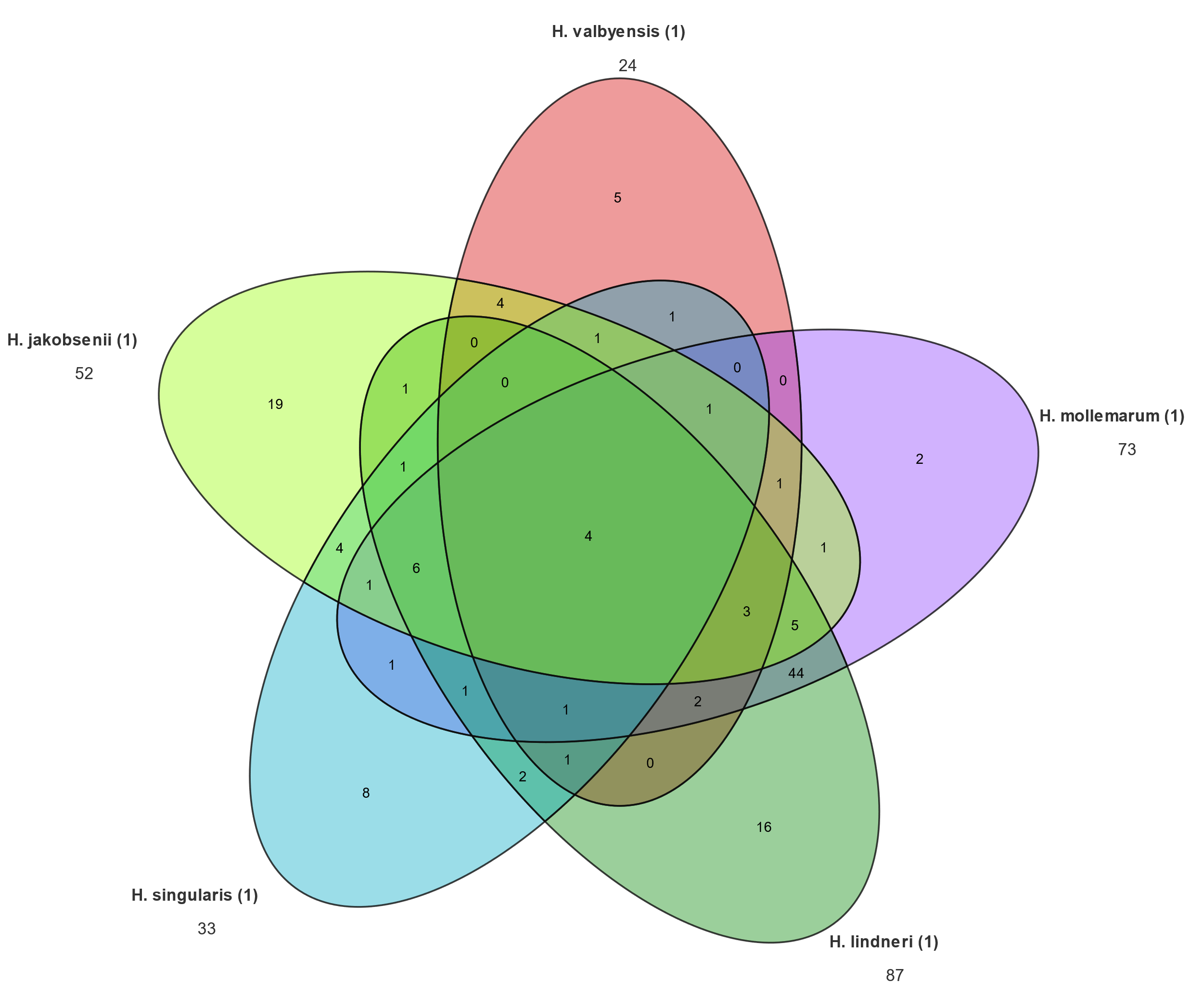


**Supplementary Figure 3A|** Venn diagrams of the genes that are lost in *H. smithiae* sp. nov. but present in its closest relatives.
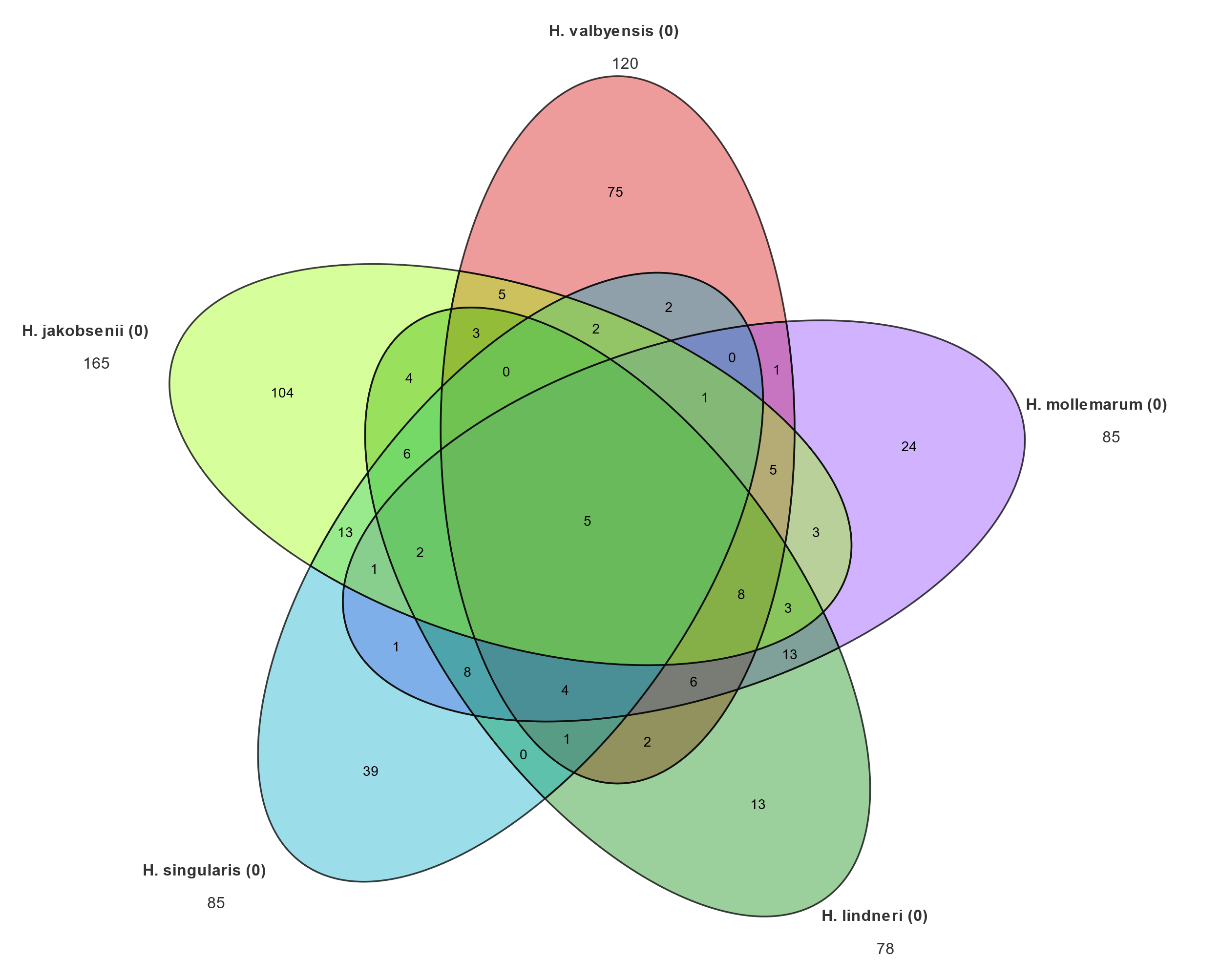


**Supplementary Figure 3B|** Venn diagrams of the genes that are present in *H. smithiae* but lost in its closest relatives.
